# Analysis of the Expression and Activity of Cyclooxygenases COX-1 and COX-2 in THP-1 Monocytes and Macrophages Cultured with Xenogenic Collagen Matrices Biofunctionalized with the Injectable Platelet-Rich Fibrin

**DOI:** 10.3390/ijms26094386

**Published:** 2025-05-05

**Authors:** Agnieszka Droździk, Katarzyna Barczak, Mateusz Bosiacki, Patrycja Kupnicka, Diana Cenariu, Willi Andrei Uriciuc, Dariusz Chlubek, Mariusz Lipski, Marek Droździk, Irena Baranowska-Bosiacka

**Affiliations:** 1 Laboratory of Preclinical Periodontology, Pomeranian Medical University in Szczecin, Powstańców Wlkp 72, 70-111 Szczecin, Poland; agnieszka.drozdzik@pum.edu.pl; 2 Department of Conservative Dentistry and Endodontics, Pomeranian Medical University in Szczecin, Powstańców Wlkp 72, 70-111 Szczecin, Poland; kasiabarczak@vp.pl; 3 Department of Biochemistry and Medical Chemistry, Pomeranian Medical University in Szczecin, Powstańców Wlkp 72, 70-111 Szczecin, Poland; mateusz.bosiacki@pum.edu.pl (M.B.); dchlubek@pum.edu.pl (D.C.); irena.baranowska.bosiacka@pum.edu.pl (I.B.-B.); 4 MEDFUTURE—Research Center for Advanced Medicine, “Iuliu Hatieganu” University of Medicine and Pharmacy, 400337 Cluj-Napoca, Romania; diana.cenariu@umfcluj.ro; 5 Faculty of Nursing and Science of Health, “Iuliu-Hatieganu” University of Medicine and Pharmacy, 400012 Cluj-Napoca, Romania; willi.uriciuc@umfcluj.ro; 6 Department of Preclinical Conservative Dentistry and Preclinical Endodontics, Pomeranian Medical University in Szczecin, Powstańców Wlkp 72, 70-111 Szczecin, Poland; lipam@pum.edu.pl; 7 Department of Experimental and Clinical Pharmacology, Pomeranian Medical University in Szczecin, Powstańców Wlkp 72, 70-111, Szczecin, Poland

**Keywords:** xenogenic collagen matrices, THP-1 monocytes, macrophages, cyclooxygenase-1 (COX-1), cyclooxygenase-2 (COX-2), inflammatory reaction, injectable platelet-rich fibrin (iPRF)

## Abstract

Xenogenic collagen matrices are used in clinical practice for soft tissue augmentation around teeth and implants, either alone or biofunctionalized with injectable platelet-rich fibrin (iPRF). Their direct interaction with inflammatory cells may influence both healing and destructive inflammation processes. Therefore, expression of cyclooxygenases (COX-1 and COX-2) and prostanoids (PGE2 and TXB2) was studied in THP-1 monocyte/macrophage cultures exposed to porcine collagen matrices (a non-cross-linked monolayer scaffold composed of collagen type I, collagen type III, and elastin (MLCM), a bilayer scaffold made of collagen types I and III (BLCM), and a volume-stable cross-linked monolayer scaffold (VSCM)). The study showed that VSCM and MLCM significantly reduced PGE2 concentrations in THP-1 monocyte cultures. iPRF further reduced PGE2 concentrations when exposed to MLCM. In contrast, incubation of THP-1 monocytes with VSCM and BLCM resulted in a significant increase in TXB2 concentrations compared with control conditions. Incubation of macrophages with MLCM, VSCM, and BLCM increased PGE2 concentrations, with VSCM and BLCM additionally increasing TXB2 concentrations. iPRF in macrophage cultures with VSCM and BLCM also resulted in increased PGE2 and TXB2 concentrations compared with control conditions. Confocal microscopy revealed no visible differences in COX-1 immunoexpression in monocytes and macrophages cultured with collagen matrices, either with or without iPFR. Weak positive COX-2 immunofluorescence was observed in monocytes, while moderate positive immunofluorescence was detected in macrophages. In conclusion, it can be suggested that the studied collagen matrices interact with monocytes/macrophages, with MLCM exhibiting the highest compatibility.

## 1. Introduction

Soft tissue augmentation procedures, aimed at enhancing the function and aesthetics of soft tissues surrounding teeth and implants, have garnered increasing attention. This trend is driven by growing aesthetic awareness among patients and the benefits these procedures offer in maintaining the long-term stability, functionality, and prognosis of both teeth and implants [1]. While autologous grafts remain the gold standard for creating and restoring adequate soft tissue dimensions [2], these procedures present notable disadvantages and limitations, primarily due to the requirement for a second surgical site at donor area and the limited availability of donor tissue for treating multiple soft tissue defects [3,4,5]. To address these challenges, research has focused on developing alternative non-autologous substitutes, with animal-derived remodeling collagen matrices (CMs) emerging as a notable example [1,6,7,8]. Several methods exist for processing xenogeneic matrices, with various elements of the nano- and microstructure of collagen playing a crucial role in guiding appropriate cellular responses [9,10].

Cell and tissue reactions can vary depending on factors such as collagen scaffold thickness, porosity, source, purification methods, and chemical cross-linking [11]. Although cross-linking enhances volume stability, it may also promote inflammatory and foreign-body reactions, potentially leading to wound healing complications and suboptimal clinical outcomes [1]. Furthermore, the slower, prolonged degradation of cross-linked collagen matrices can result in residues and secondary products with potentially toxic effects [3,12]. To minimize immunogenicity, the manufacturing process for collagen matrices eliminates cells, proteins, and non-protein substances from the original tissue. As a result, collagen matrices lack cell-inducing and tissue-regenerating capabilities, features that are highly desirable in soft tissue augmentation procedures [13].

Wound healing, along with soft and hard tissue regeneration, can be enhanced by injectable PRF (iPRF). Developed through centrifugation of blood in non-glass centrifugation tubes at lower speeds and for shorter durations [14], iPRF represents the latest and most significant advancement in platelet concentrates. Following application, liquid iPRF gradually transforms into a three-dimensional fibrin network that embeds platelets, leukocytes, type I collagen, osteocalcin, and growth factors. These bioactive components are continuously released over a period of 10–14 days, promoting wound healing and supporting both soft and hard tissue regeneration. iPRF is widely used in regenerative dentistry as an injectable biomaterial, a carrier for various biomolecules, or in combination with other biomaterials for diverse clinical applications [15]. Combining injectable platelet-rich fibrin (iPRF) with collagen scaffolds provides clinically relevant benefits, such as enhanced cell proliferation. Collagen membranes soaked in iPRF significantly boost gingival mesenchymal stem cell attachment, penetration depth, and proliferation [16]. Collagen membranes biofunctionalized with iPRF, used in a split-mouth randomized trial employing the minimally invasive VISTA technique, showed significantly greater improvements in clinical parameters related to root coverage, making the procedure more predictable [17]. It can be hypothesized that the bioactive molecules in iPRF, when applied to collagen matrices, may contribute to creating a more favorable environment for surrounding cells during the healing process.

The pathophysiology of oral tissue healing is highly complex and not yet fully understood. This biological process involves numerous cell-to-cell and cell-to-extracellular matrix interactions, regulated by signaling protein molecules such as chemokines, cytokines, and growth factors. It occurs in four overlapping phases: hemostasis, inflammation, proliferation, and remodeling [18,19]. The use of absorbable devices, following a critical early healing sequence, inevitably elicits tissue reactions that may influence wound healing. Ideally, these inflammatory reactions should be limited in magnitude, tolerable in nature, and should not compromise the regeneration outcome. Currently, various materials with advanced biochemical properties are employed in the treatment of soft tissue deficiencies. Their composition is continually refined to mimic the characteristics of autologous soft tissue grafts. However, despite the ongoing development of new materials and processing techniques, certain side effects persist, such as the triggering of an immune reaction, that may exacerbate inflammation. This heightened in inflammatory state can lead to early exposure and premature degradation of collagen matrices [20]. In light of previous preclinical and clinical studies [3,7,21,22,23], collagen matrices appear to be biocompatible materials. Nevertheless, clinical evaluations alone cannot fully elucidate the underlying biological mechanisms, including the local inflammatory response.

Monocytes and macrophages are among the first cells to interact with the implanted materials immediately after their placement on exposed or partially damaged tissue [24]. These cells, which possess the ability to synthesize and secrete cytokines, serve as a key source of these signaling molecules. This characteristic, among other factors, explains their role in initiating both destructive and reparative processes [25]. Inflammation is closely associated with the activity of macrophages that participate in response to various stimuli, including materials such as collagen matrices [11,26,27]. Through the secretion of cytokines and growth factors, macrophages initiate both reparative and destructive processes [27,28]. The initial phase following implantation is characterized by the intensive secretion of pro-inflammatory cytokines, including IL-1β, IL-6, and TNF-α, as well as chemokines such as monocyte chemoattractant protein-1 (MCP-1) and macrophage inflammatory protein-1α (MIP-1α) [28,29,30,31]. Studies have also shown that, under in vitro conditions, macrophages increase the expression and activity of cyclooxygenase-1 (COX-1) and cyclooxygenase-2 (COX-2). These enzymes catalyze the conversion of arachidonic acid into prostaglandin H_2_ (PGH2). This process occurs in two stages, with PGH2 serving as a precursor for biologically active prostanoids such as prostaglandin E2 (PGE2) and thromboxane A_2_ (TXA_2_) [32,33]. Until recently, COX-1 was considered a constitutive enzyme with minimal involvement in the inflammatory process [34]. However, it is now recognized that, in certain tissues, this enzyme is sensitive to induction and plays a role in the initial phase of the response to factors initiating prostanoid synthesis. COX-1 is a source of PGE2 and TXA_2_, the latter being metabolized into the more stable TXB2. COX-2 is an enzyme that undergoes induced expression in response to factors such as pro-inflammatory cytokines or cytokines produced by cell growth factors (including monocytes) and plays a dominant role in chronic inflammation [35,36]. In monocytes, COX-1 activity predominantly supports TXA_2_ production, while COX-2 activity favours PGE2 production [37].

The aim of this study was to investigate whether three different porcine collagen matrices, routinely used for soft tissue augmentation around teeth and implants, both alone and biofunctionalized with iPRF, influence the expression of COX-1 and COX-2 enzymes in THP-1 monocytes/macrophages. Additionally, their effect on the synthesis of prostanoids (PGE2 and TXB2), which serve as mediators of inflammation and are catalyzed by COX-1 and COX-2, was evaluated. This experimental approach enables the replication of clinical conditions resulting from the direct interaction between collagen matrices and soft tissue, mimicking the processes occurring during healing and regeneration in augmentation procedures.

## 2. Results

### 2.1. Collagen Matrices Induced Activation of THP-1 Monocytes

**Figure 1:**
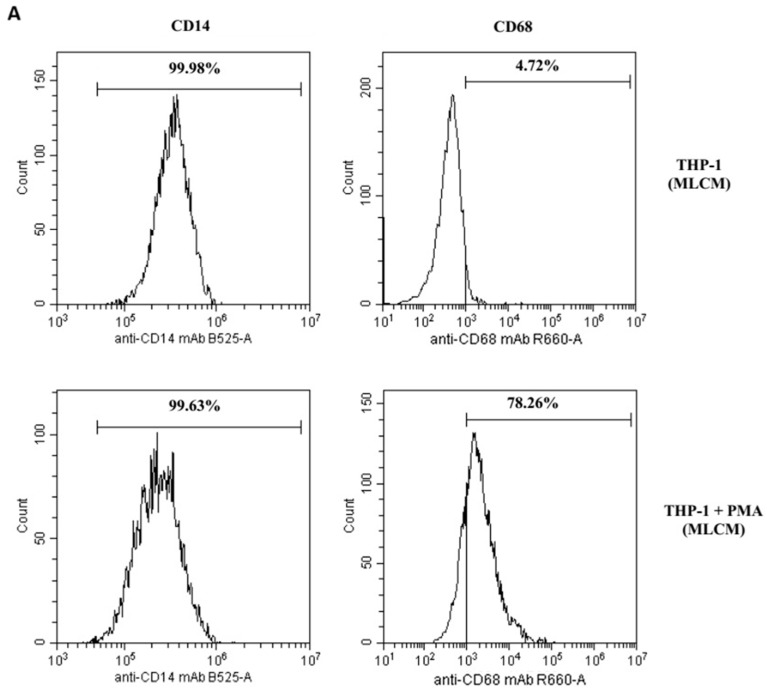

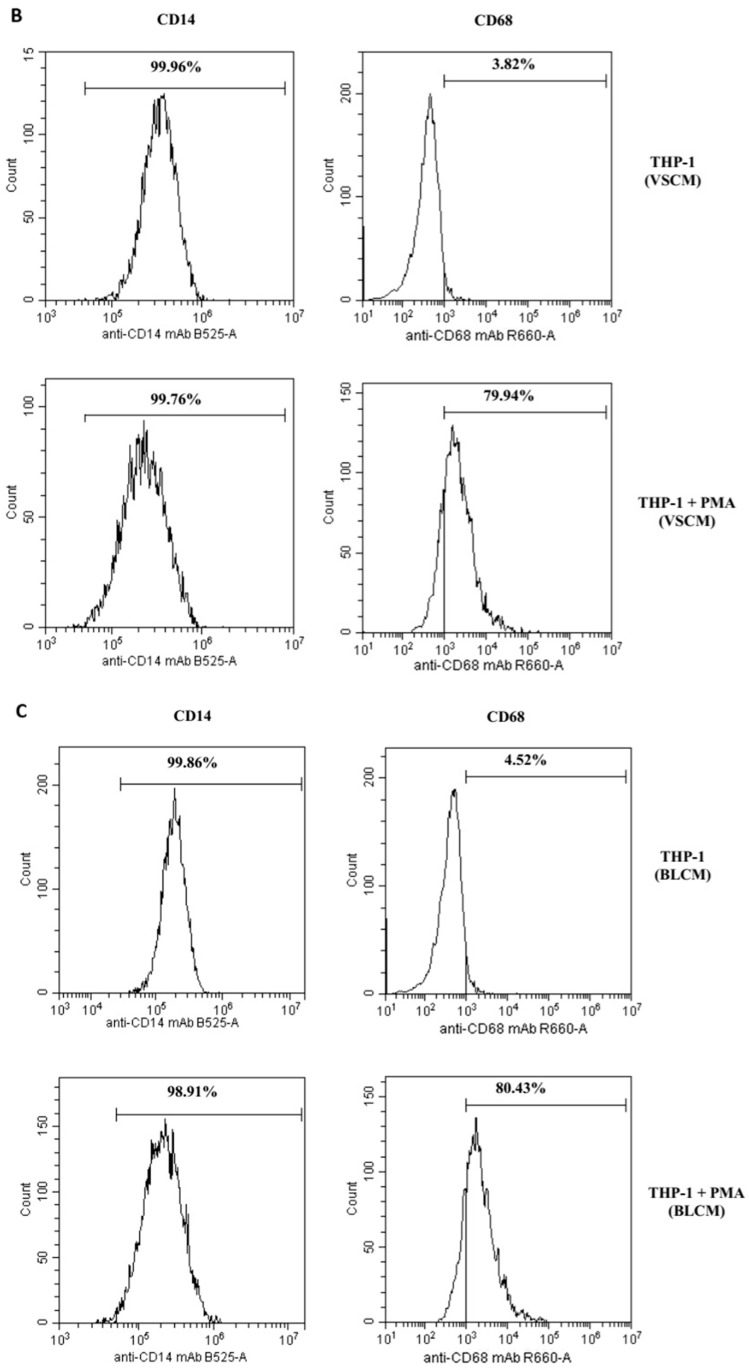
The effect of the collagen matrices (**A**) MLCM, (**B**) VSCM, and (**C**) BLCM on the differentiation of THP-1 monocytes to macrophages. The THP-1 cells in the presence of collagen matrices were incubated without phorbol 12-myristate 13-acetate (PMA, upper histograms) or with PMA (lower histograms). Expression of markers was determined by flow cytometry. CD68 expression (marker for macrophages) increased significantly after treatment with PMA (200 nM) as compared with non-treated cells, whereas CD14 expression (marker for monocytes) did not change. The figure shows representative histograms obtained by flow cytometry.

All tested collagen matrices induced activation of THP-1 monocytes, as demonstrated by an increase in CD68 expression (a macrophage differentiation marker) after 48 h of treatment with PMA (200 nM) compared with untreated cells. The expression was measured by flow cytometry. In contrast, the expression of CD14 (a monocyte differentiation marker) remained unchanged (see Table 1, and Figure 1A–C).

### 2.2. THP-1 Monocyte and Macrophage Culture Visualization

**Figure 2:**
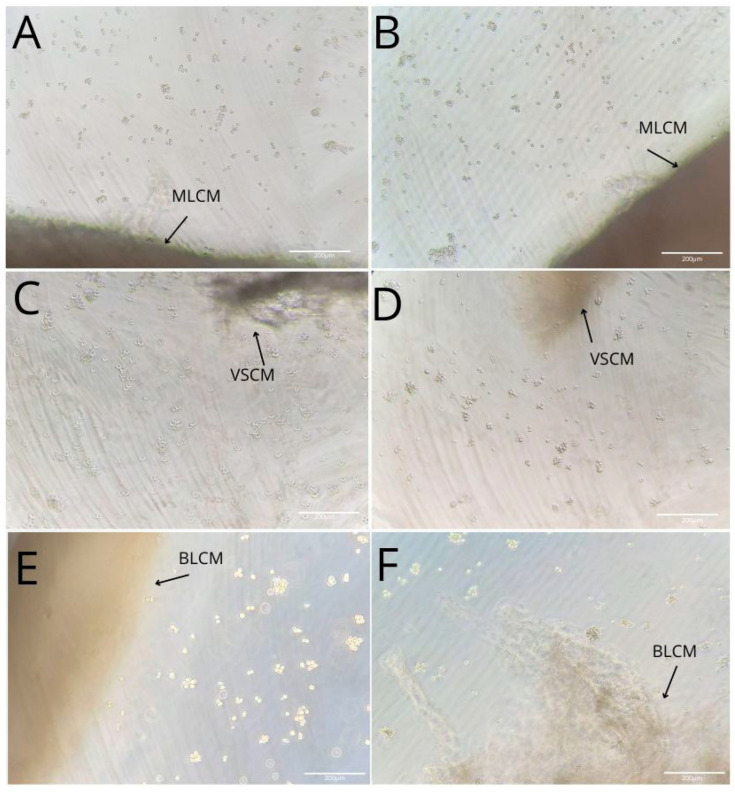
Imaging of THP-1 monocyte (**A**,**C**,**E**) and macrophage (**B**,**D**,**F**) cultures with MLCM (**A**,**B**), VSCM (**C**,**D**), and BLCM (**E**,**F**).

**Figure 3:**
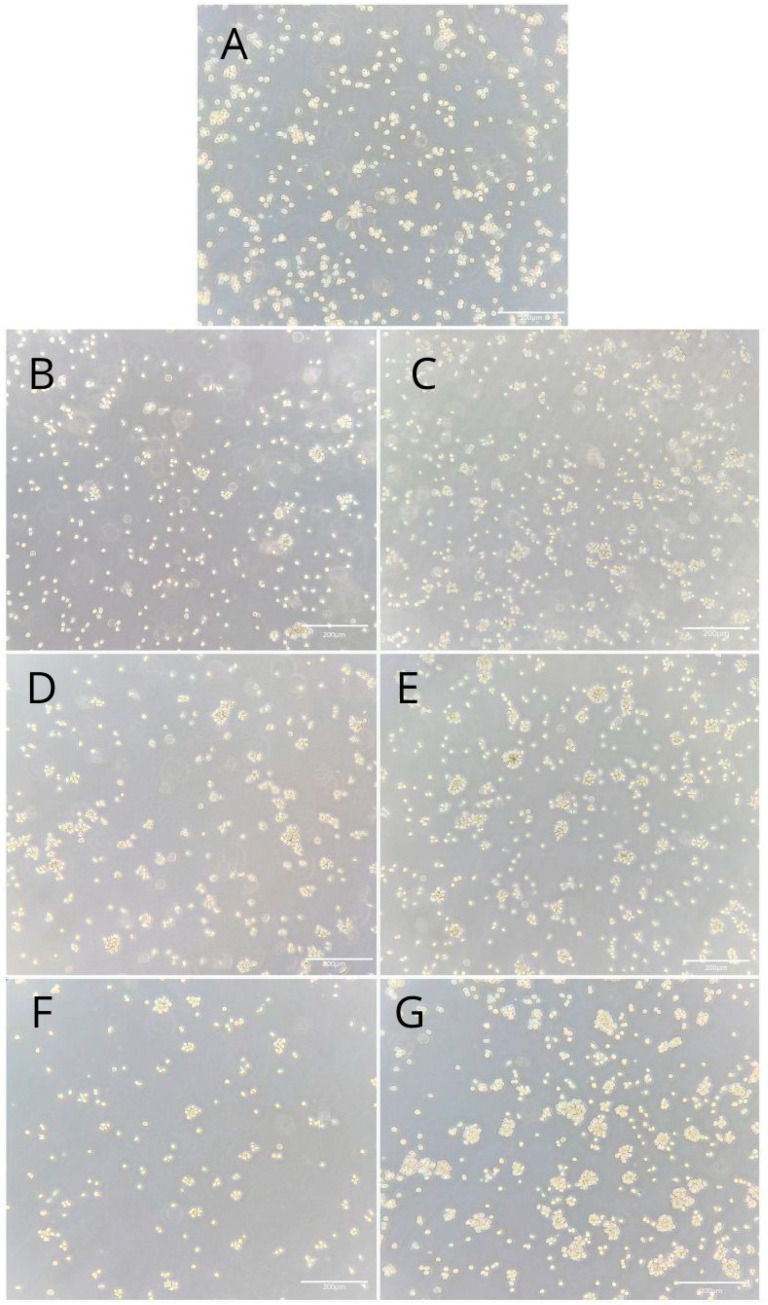
Imaging of THP-1 monocyte cultures. Cells were cultured in RPMI medium with 10% FBS as a control condition for 48 h (**A**), and under the following conditions: MLCM (**B**), MLCM + iPRF (**C**), VSCM (**D**), VSCM + iPRF (**E**), BLCM (**F**), and BLCM + iPRF (**G**). Normal cell morphology was observed across all conditions. The experiments were performed in six separate assays, each conducted in triplicate.

**Figure 4:**
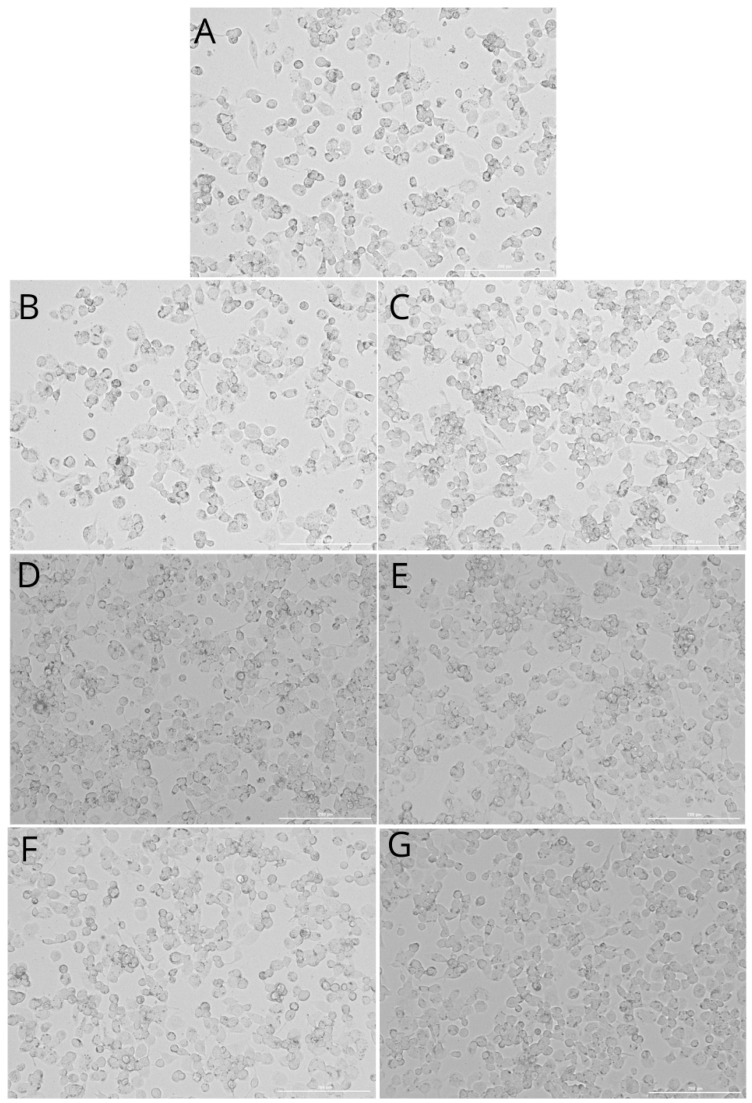
Imaging of the macrophage culture. Cells were cultured in RPMI medium with 10% FBS as a control condition for 48 h (**A**), and under the following conditions: MLCM (**B**), MLCM + iPRF (**C**), VSCM (**D**), VSCM + iPRF (**E**), BLCM (**F**), and BLCM + iPRF (**G**). Normal cell morphology was observed in all conditions. Experiments were conducted as six separate assays, with each assay performed in triplicate.

Monocytes and macrophages cultured under control conditions (RPMI medium with 10% FBS) for 48 h, as well as those incubated with MLCM, VSCM, and BLCM with or without iPRF for 48 h, showed normal morphology (Figure 2, Figure 3 and Figure 4).

### 2.3. Prostaglandin E2 (PGE2) in THP-1 Monocytes and Macrophages

**Figure 5:**
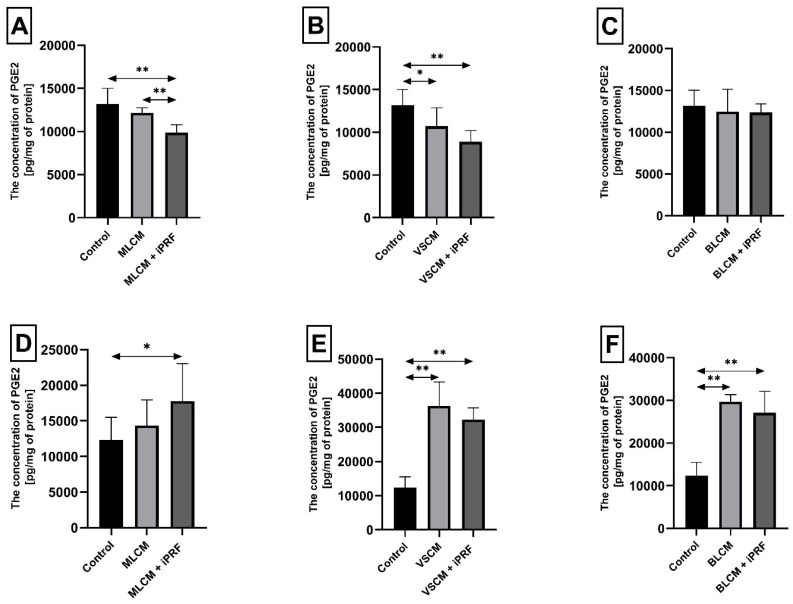
Prostaglandin E2 (PGE2) concentration in THP-1 monocytes (**A**–**C**) and macrophages (**D**–**F**) incubated with collagen matrices (MLCM, VSCM, and BLCM) with or without injectable platelet-rich fibrin (iPRF). Cells were cultured for 48 h in RPMI medium supplemented with 10% FBS. Following incubation, cells were harvested by scraping, and PGE2 concentrations were measured by ELISA. Control cells were cultured in RPMI medium with 10% FBS without collagen matrices. The experiments were conducted as six independent assays, each conducted in triplicate. * indicates a statistically significant difference compared with the control (*p* ≤ 0.05), while ** denotes a highly significant difference compared with the control (*p* ≤ 0.01), as determined by a U-Mann–Whitney test.

Incubation of THP-1 monocytes under MLCM + iPRF conditions for 48 h resulted in a statistically significant decrease in PGE2 concentration in the medium by approximately 25% compared with the control condition (*p* = 0.002). Furthermore, monocytes incubated with MLCM + iPRF showed an 18.7% reduction in PGE2 concentration (*p* = 0.002) compared with those treated with MLCM alone. Similarly, monocytes incubated with VSCM showed a 19% reduction in PGE2 concentration compared with control conditions (*p* = 0.002), while VSCM + iPRF further decreased PGE2 levels by 33% compared with the control (*p* = 0.002). In contrast, monocytes incubated with BLCM and BLCM + iPRF did not show statistically significant changes in PGE2 concentration (Figure 5A–C).

Macrophages incubated with MLCM + iPRF for 48 h exhibited a statistically significant increase in PGE2 concentration by 44% compared with control conditions (*p* = 0.002). Similarly, macrophages cultured with VSCM showed a dramatic increase in PGE2 concentration by 194% relative to control conditions (*p* = 0.002). A comparable elevation in PGE2 concentration (161%, *p* = 0.002) was observed in macrophages incubated with VSCM + iPRF. Macrophages incubated with BLCM also demonstrated a substantial rise in PGE2 concentration by 140% compared with the control (*p* = 0.002). In the presence of BLCM + iPRF, PGE2 levels increased by 119% (*p* = 0.002) (Figure 5D–F).

### 2.4. Thromboxane TXB2 in THP-1 Monocytes and Macrophages

**Figure 6:**
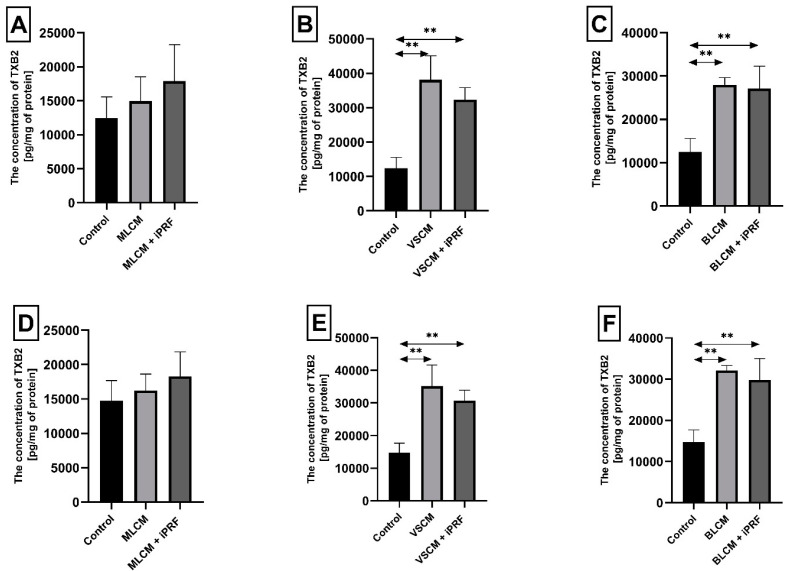
Thromboxane 2 (TXB2) concentration in THP-1 monocytes (**A**–**C**) and macrophages (**D**–**F**) cultured with collagen matrices (MLCM, VSCM, and BLCM) with or without injectable platelet-rich fibrin (iPRF). Cells were cultured for 48 h in RPMI medium supplemented with 10% FBS and collagen matrices. Following incubation, cells were harvested by scraping, and TXB2 concentration in the medium was measured by ELISA. Control cells were cultured in RPMI medium with 10% FBS without collagen matrices. Experiments were conducted in six independent assays, each performed in triplicate. ** Indicates a statistically significant difference compared with the control ((*p* ≤ 0.01) (U-Mann–Whitney test)).

Incubation of THP-1 monocytes for 48 h under VSCM conditions caused a statistically significant increase in thromboxane TXB2 concentration in the cell medium by approximately 206% compared with control conditions (*p* = 0.002). Similarly, monocytes incubated with VSCM + iPRF exhibited a 160% higher TXB2 concentration compared with the control (*p* = 0.002). In culture treated with BLCM, the TXB2 concentration increased by 125% relative to control conditions (*p* = 0.002), while cells incubated with BLCM + iPRF showed a 118% increase TXB2 concentration compared with the control (*p* = 0.002) (Figure 6A–C).

Macrophages cultured with VSCM for 48 h exhibited a statistically significant increase in TXB2 concentration in the medium by 138% compared with control conditions (*p* = 0.002). In macrophages treated with VSCM + iPRF, TXB2 levels were 108% higher than the control (*p* = 0.002). Similarly, macrophages incubated with BLCM showed an approximately 118% increase in TXB2 concentration compared with the control conditions (*p* = 0.002). Those treated with BLCM + iPRF exhibited a 102% higher TXB2 concentration relative to the control (*p* = 0.002) (Figure 6D–F). Other observed changes in thromboxane concentration in macrophage cultures were not statistically significant.

### 2.5. Cyclooxygenase-1 and Cyclooxygenase-2 Expression in Monocytes and Macrophages

**Figure 7:**
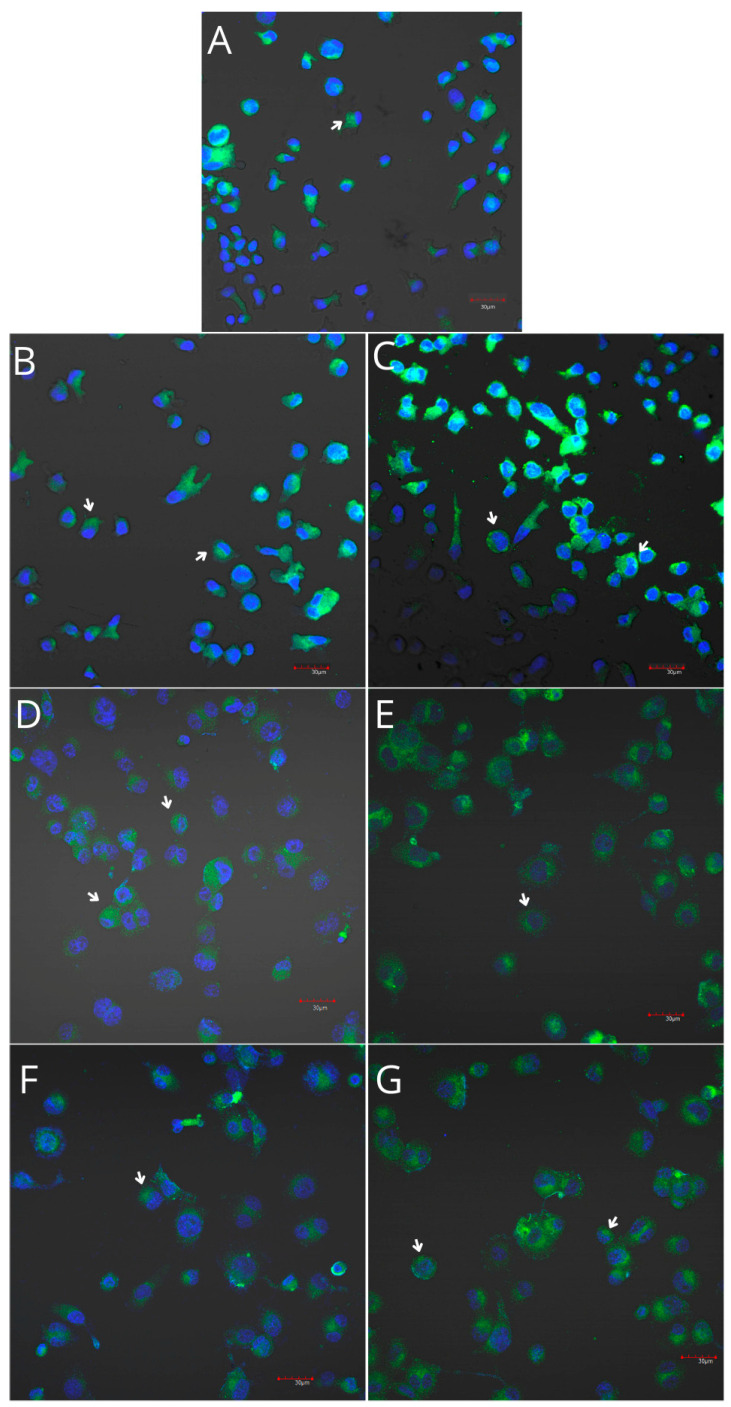
Imaging of COX-1 enzyme expression in THP-1 monocytes by fluorescence microscopy (I.) and representative Western blots (II.). Cells were cultured in RPMI medium with 10% FBS as the control condition for 48 h (**A**) and under the following conditions: MLCM (**B**), MLCM + iPRF (**C**), VSCM (**D**), VSCM + iPRF (**E**), BLCM (**F**), and BLCM + iPRF (**G**). The immunohistochemistry was performed using a specific primary antibody (mouse anti-COX-1 incubated overnight at 4 °C) and secondary antibodies conjugated with FITC (anti-mouse IgG-FITC, incubated for 45 min at room temperature). COX-1 expression is visible as green fluorescence, while cell nuclei were stained with DAPI (blue). Confocal microscopy was used for image analysis, employing filters 38 HE GFP for green fluorescence and 49 DAPI for blue fluorescence. Image analysis was performed with a confocal microscope using filters 38 HE GFP for green fluorescence and 49 DAPI for blue fluorescence. Arrows indicate areas of increased COX-1 expression compared with the control.

**Figure 8:**
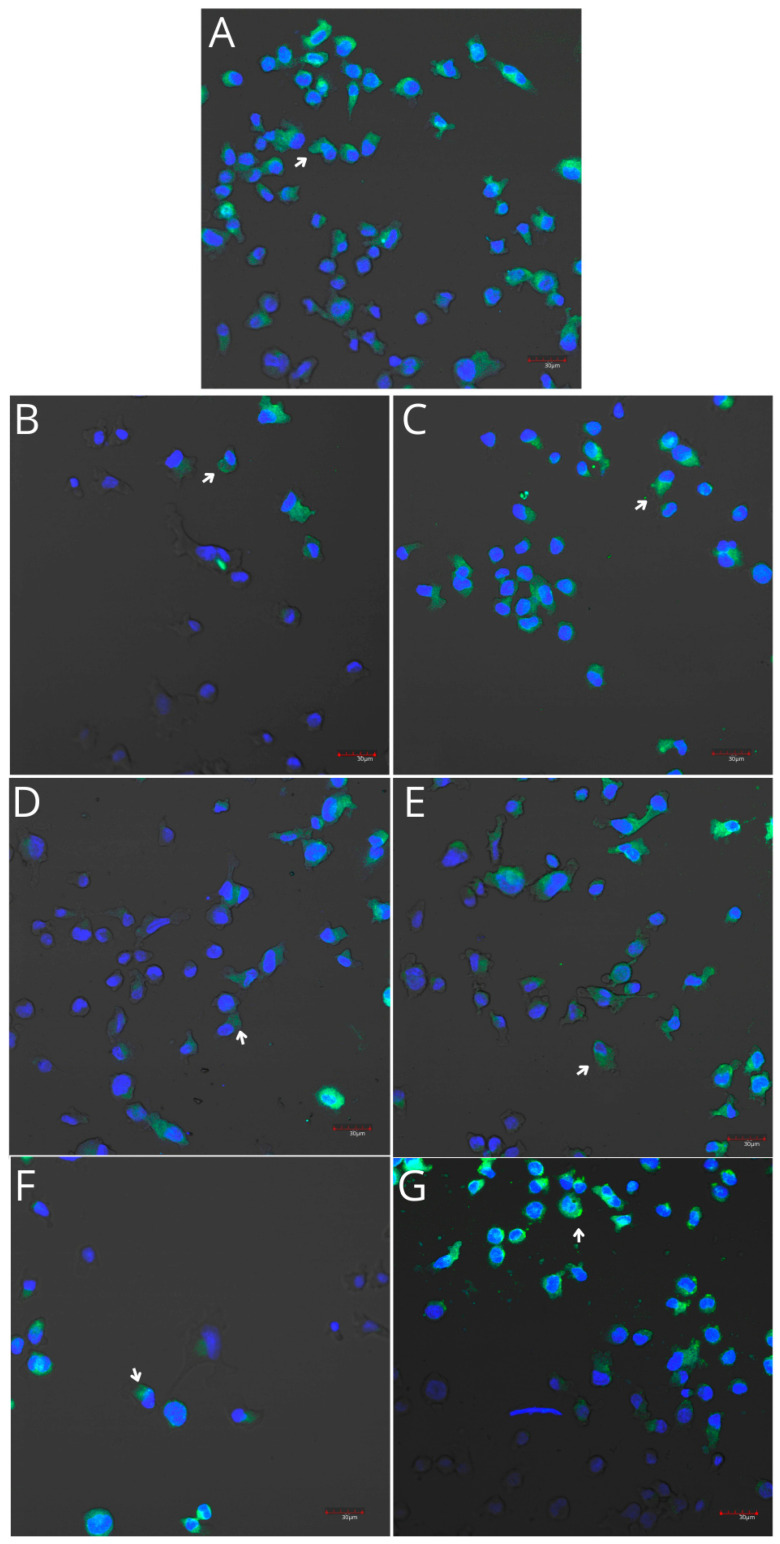
Imaging of COX-2 enzyme expression in THP-1 monocytes by fluorescence microscopy (I.) and representative Western blots (II.). Cells were cultured in RPMI medium with 10% FBS as the control condition for 48 h (**A**) and under the following conditions: MLCM (**B**), MLCM + iPRF (**C**), VSCM (**D**), VSCM + iPRF (**E**), BLCM (**F**), and BLCM + iPRF (**G**). Immunohistochemistry was performed using specific primary antibodies (mouse anti-COX-2, incubated overnight at 4 °C) and secondary antibodies conjugated with FITC (anti-mouse IgG-FITC, incubated for 45 min at room temperature). COX-2 expression is visible as green fluorescence, while cell nuclei were stained with DAPI (blue). A confocal microscope was used for image analysis, employing filters 38 HE GFP for green fluorescence and 49 DAPI for blue fluorescence. Arrows indicate areas of increased COX-2 expression compared with the control.

**Figure 9:**
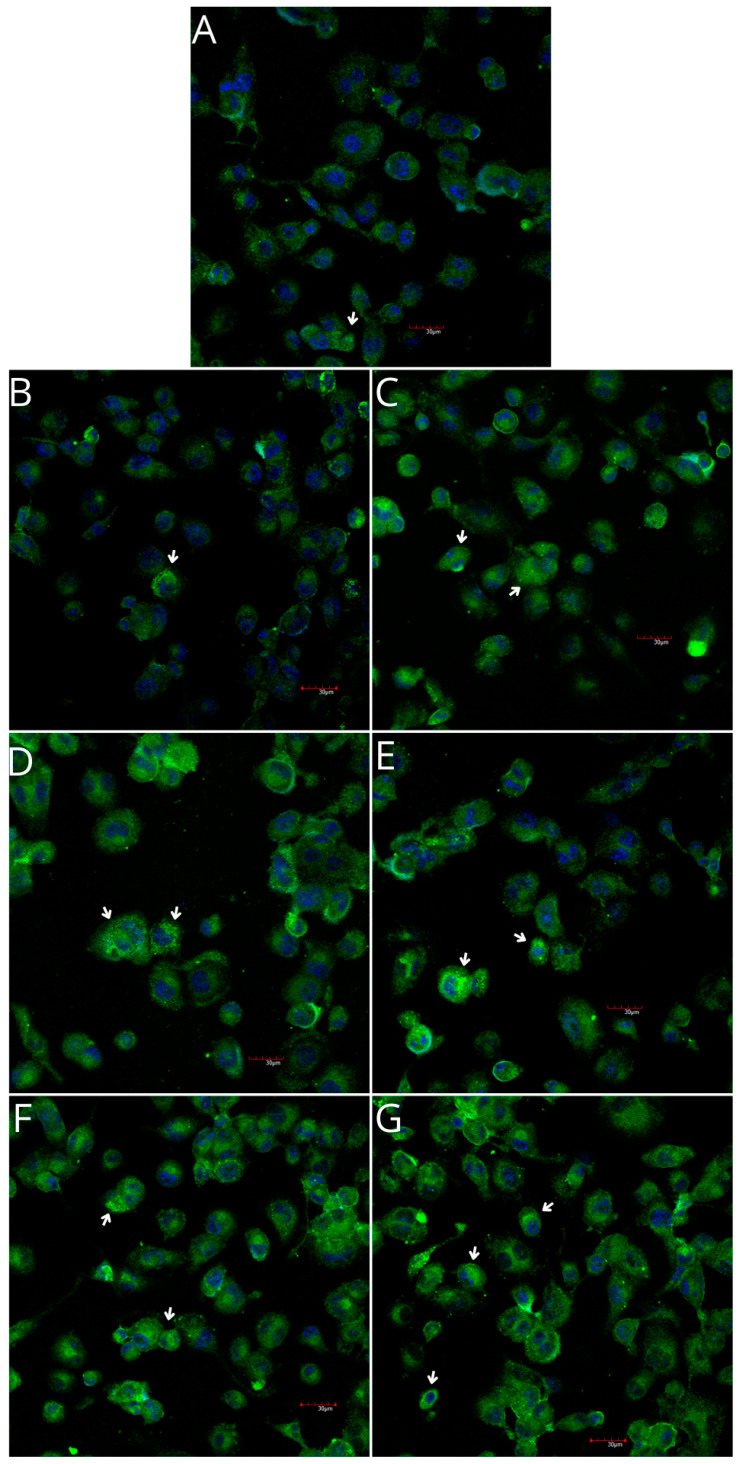
Imaging of COX-1 enzyme expression in THP-1 macrophages by fluorescence microscopy (I.) and representative Western blots (II.). Cells were cultured in RPMI medium with 10% FBS as the control condition for 48 h (**A**) and under the following conditions: MLCM (**B**), MLCM + iPRF (**C**), VSCM (**D**), VSCM + iPRF (**E**), BLCM (**F**), and BLCM + iPRF (**G**). Immunohistochemistry was performed using specific primary antibodies (mouse anti-COX-1) and secondary antibodies conjugated with FITC (anti-mouse IgG-FITC, incubated for 45 min at room temperature). COX-1 expression is visualized as green fluorescence, while cell nuclei were stained with DAPI (blue). Confocal microscopy was used for image analysis, employing filters 38 HE GFP for green fluorescence and 49 DAPI for blue fluorescence. Arrows indicate areas of increased COX-1 expression compared with the control.

**Figure 10:**
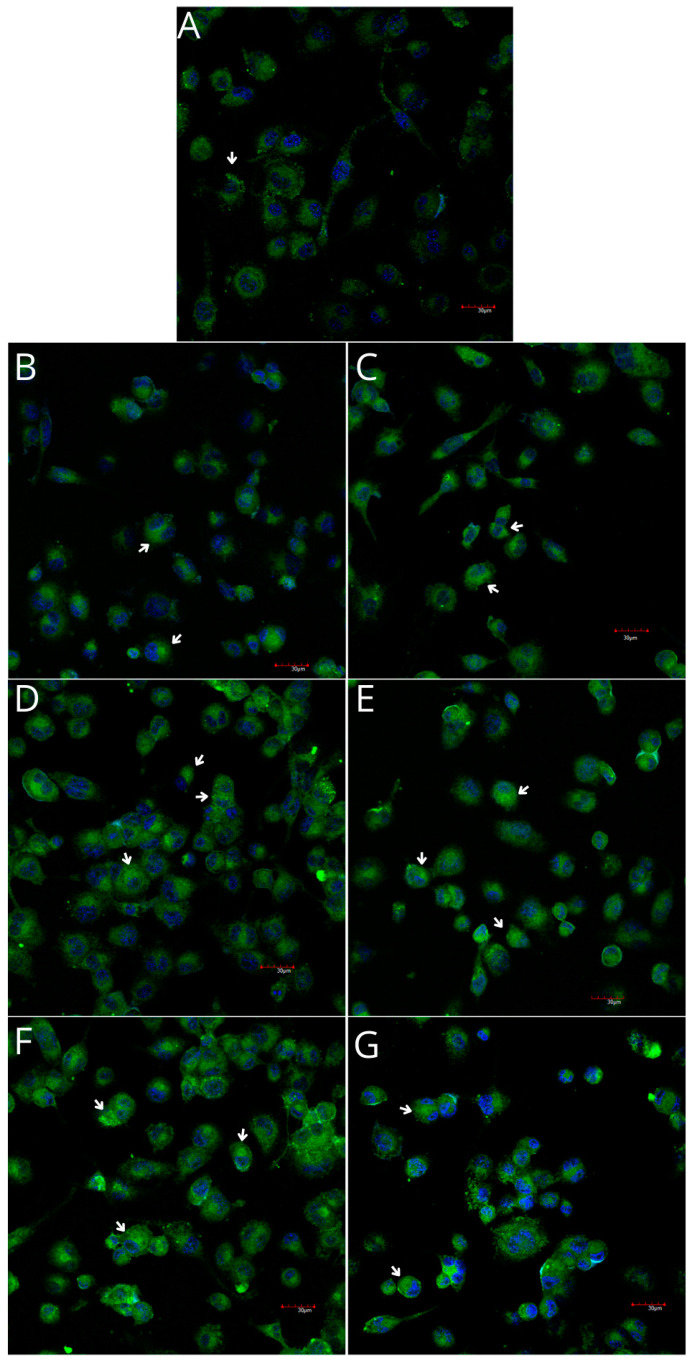
Imaging of COX-2 enzyme expression in THP-1 macrophages by fluorescence microscopy (I.) and representative Western blots (II.). Cells were cultured in RPMI medium with 10% FBS as the control condition for 48 h (**A**) and under the following conditions: MLCM (**B**), MLCM + iPRF (**C**), VSCM (**D**), VSCM + iPRF (**E**), BLCM (**F**), and BLCM + iPRF (**G**). Immunohistochemistry was performed using specific primary antibodies (mouse anti-COX-2) and secondary antibodies conjugated with FITC (anti-mouse IgG-FITC, incubated for 45 min at room temperature). COX-2 expression is visualized as green fluorescence, and cell nuclei were stained with DAPI (blue). Confocal microscopy was used for image analysis, employing filters 38 HE GFP for green fluorescence and 49 DAPI for blue fluorescence. Arrows indicate areas of increased COX-2 expression compared with the control.

THP-1 monocytes and macrophages cultured with collagen matrices with or without iPRF exhibited weak positive immunofluorescence to COX-1. Confocal microscopy revealed no visible differences in COX-1 immunoexpression in monocytes and macrophages cultured with collagen matrices, with or without iPRF, compared with the control. However, weak positive COX-2 immunofluorescence was observed in monocytes, while macrophages displayed moderate positive immunofluorescence when incubated with collagen matrices, with or without iPRF, compared with the control (Table 2, Figure 7, Figure 8, Figure 9 and Figure 10).

**Figure 11:**
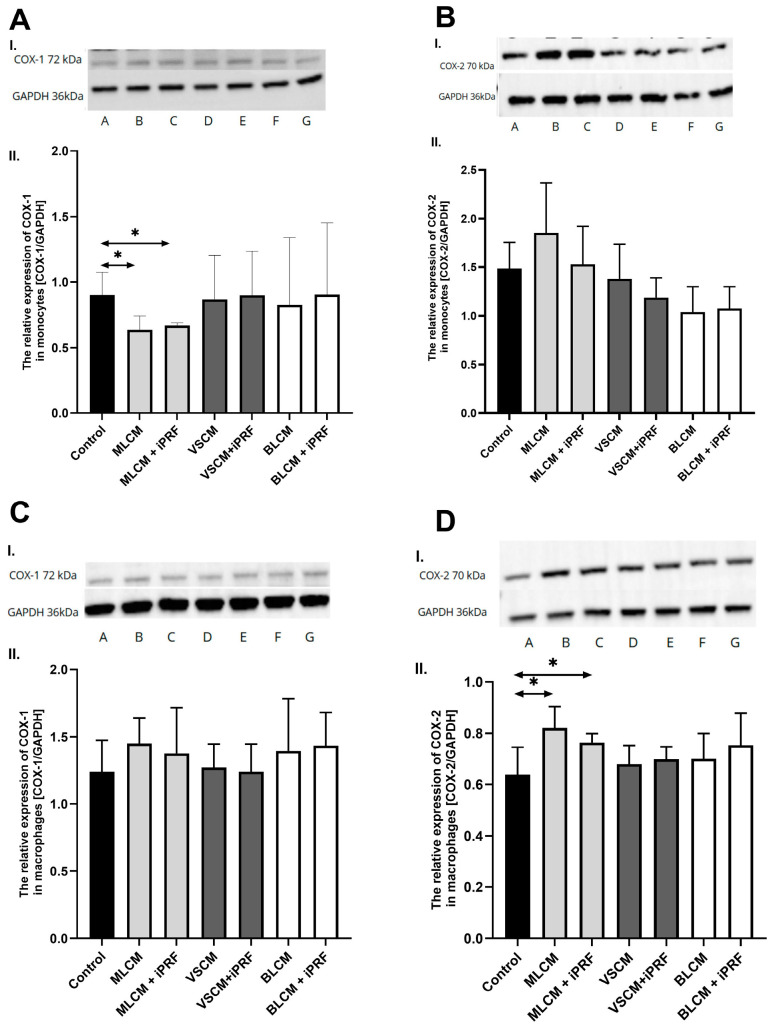
Representative blots (I.) and densitometric analysis (II.) of COX-1 (**A**,**C**) and COX-2 (**B**,**D**) protein expression in THP-1 monocytes (**A**,**B**) and macrophages (**C**,**D**). Cells were cultured in RPMI medium with 10% FBS as the control condition for 48 h and under the following conditions: MLCM and MLCM + iPRF, VSCM and VSCM + iPRF as well as BLCM and BLCM + iPRF. Data represent the mean ± standard deviation. * *p* < 0.01 (Mann–Whitney U test).

In Western blot analysis, THP-1 monocytes and macrophages cultured with collagen matrices with or without iPRF exhibited weak positive immunofluorescence to COX-1. In the MLCM and MLCM + iPRF groups of monocytes, the expression of COX-1 was significantly lower compared with the control group (*p* = 0.01; *p* = 0.01, respectively). There were no visible differences in COX-1 immunoexpression in macrophages cultured with collagen matrices, with or without iPRF, compared to the control. There were no significant differences in COX-2 immunofluorescence between the studied groups in monocytes, while in the macrophages the MLCM and MLCM + iPRF groups had significantly higher expression of COX-2 than the control group (*p* = 0.01; *p* = 0.01, respectively) (Figure 11).

## 3. Discussion

Data on the cellular and molecular mechanisms associated with collagen matrices remain limited and often contradictory [31,38]. On the other hand, understanding these mechanisms is of great interest to materials scientists and clinicians as it could enhance the predictability of collagen matrices in soft tissue regeneration. Previous animal studies, where collagen matrices were implanted subcutaneously, have demonstrated variable cellular responses depending on the material’s specific characteristics and manufacturing techniques. These responses ranged from mononuclear cell infiltration (e.g., macrophages) without a foreign-body reaction to multinucleated giant cell-induced granulation tissue formation [3,11,24].

In an in vitro study with porcine- and bovine-derived collagen membranes, all tested membranes—particularly the porcine-derived ones—induced increased production of pro-inflammatory mediators in the mononuclear cells and decreased cellular proliferation, as measured by the MTT assay [31]. These findings contrast with the results of Liu et al., who studied the effects of collagen membranes on hMSC proliferation using lactate dehydrogenase and MTT assays. In their study, porcine-derived collagen membranes exhibited low cytotoxicity and significantly enhanced cellular proliferation compared with controls [38].

In the present study, we investigated the effects of collagen matrices, both alone and biofunctionalized with iPRF, on the expression of COX-1 and COX-2 enzymes in THP-1 monocytes/macrophages as well as on the synthesis of prostanoids (PGE2) and tromboxanes (TXB2). The THP-1 cell line, including both monocytes and the macrophages derived from them, represents a widely used cellular model for studying the immune response. THP-1 cells closely mimic human monocytes/macrophages in terms of morphology and function [39].

This study provides information about the collagen matrix interactions at a single time point, without demonstrating time-dependent characteristics, which may be considered a study limitation. However, since collagen matrices are intended to support wound healing—a process involving four sequential but overlapping phases: hemostasis (0 to several hours after injury), inflammation (1–3 days), proliferation (4–21 days), and remodeling (21 days to 1 year) [40]—the focus of the present study is justified. The functions of the monocytes/macrophages assessed in the present study are primarily involved in the inflammatory phase; therefore, a 48 h time point was chosen to evaluate the interactions between these cells and the collagen matrices.

In the literature, THP-1 cells are described as an appropriate model for studying monocyte/macrophage responses, determining macrophage differentiation, and analyzing the effects of external factors on macrophages [39]. The results obtained using these cells can often be extrapolated to the human organism. The same in vitro study model was used by Sikora et al. to determine how the production of PGE2 and TXB2 in THP-1 monocytes/macrophages is influenced by titanium 3D plates and dedicated screws [41]. This model was also utilized in a study aimed at investigating whether silicate-based materials, used in the regeneration of the pulp–dentine complex, affect the expression of the enzymes COX-1 and COX-2 in THP-1 monocytes/macrophages as well as the synthesis of prostanoids by these enzymes [42].

In the present study, THP-1 cells expressing a low level of CD14+ developed into the CD68+ phenotype under PMA stimulation, signifying their differentiation into mature macrophages. The macrophage phenotype evolves as the wound heals, transitioning from the M1 (pro-inflammatory) to the M2 (anti-inflammatory) form, a process influenced by the local microenvironment. The M2 phenotype is characterized by distinct surface receptors or cell differentiation (CD) markers, including CD68 [43]. This phenotype may result from the conversion of M1 macrophages—the classical activated form involved in pathogen phagocytosis and the clearance of damaged cells, including neutrophils—into M2 macrophages, which perform repair and regeneration functions [44]. The balance between M1 and M2 macrophages plays a crucial role in progression of various inflammatory diseases and the wound healing process. However, in the present study, the collagen matrices demonstrated a substantially lower induction potential compared with the model differentiation stimulator (PMA). Under the experimental conditions, a pool of macrophages with CD14+ classical monocytes was observed. The differentiation potential of THP-1 cells exposed to all studied porcine collagen matrices—a non-cross-linked monolayer scaffold of collagen types I and III and elastin (MLCM), a bilayer scaffold of collagen types I and III (BLCM), and a cross-linked monolayer scaffold of collagen types I and III and elastin with volume stability (VSCM)—was similar and of low intensity. These results suggest that the studied collagen matrices have a limited capacity to promote macrophage differentiation and, by extension, wound healing stimulation.

In the next step, the expression and activity of COX-1 and COX-2 in monocytes and macrophages were analysed. COX-1 is a housekeeping enzyme involved in maintaining the basal PG levels necessary for tissue and cell homeostasis [45]. On the other hand, COX-2 is tightly regulated, with its expression and activation directly induced by pro-inflammatory cytokines and growth factors that activate intracellular-inflammation-related pathways [46]. However, COX-1 can also participate in inflammatory responses, as demonstrated in studies on COX-1 knockout mice [36,47]. Moreover, up-regulated COX-1 expression has been observed in resident inflammatory cells responsible for acute inflammatory responses and cell differentiation [48]. In the experimental model used, monocytes expressed lower expression levels of both COX-1 and COX-2 compared with macrophages, and exposure of the cells to the studied collagen matrices did not affect the levels of these enzymes. However, semi-quantitative fluorescence microscopy may not be sensitive enough to detect subtle differences resulting from stimulation, as the levels of PGE2 and TXB2, products of COX-1 and COX-2 activity, were affected. PGE2 and TXA2 are derived from the common intermediate product PGH2. Their relative rates of production in different cell types can depend on the relative efficiencies with which their respective terminal synthases engage PGH2 and convert it into their specific eicosanoid products.

Studies on rat peritoneal cells revealed that COX-2 activity favored the production of PGE2, or prostacyclin, whereas COX-1 activity favored the production of TXA2. This finding suggests compartmentalization or a functional linkage of COX isozymes with different terminal synthases [49]. In the experimental model, TXB2 was used as a marker for biologically active TXA2. TXB2 is the chemically stable and biologically inactive hydrolysis product of TXA2 and is used as surrogate marker for TXA2 [50]. The results of the present study suggest that macrophages respond more significantly to xenogenic collagen matrices, with increased production of both PGE2 and TXB2. The fold-change for BLCM and VSCM exceeded 2, indicating biological significance. These two biomaterials appear to be less biocompatible, with a higher inflammation-induction potential compared with MLCM. These findings corroborate experimental results in rats, which demonstrated rapid tissue integration and vascularization with minimal to no signs of ingrowing inflammatory cells in native, non-cross-linked collagen. In contrast, cross-linked collagen matrices led to limited tissue integration and were associated with a higher degree of inflammatory response [3].

All the analyzed collagen matrices are derived from porcine tissues and are com-posed of collagen types I and III and/or elastin, so that potential interspecies differences can be ruled out. The observed differences can rather be attributed to physicochemical properties resulting from cross-linking density and the three-dimensional structure. These parameters can affect the stability of the collagen network (volume stability), the cell interaction surface, and the ingrowth space (e.g., for monocytes and macrophages) as well as cell behavior (including monocyte and macrophage polarization) and the degradation rate, which have been shown to influence physiology [29,51,52]. Porous matrices and those with lower stiffness, which replicate the biomechanical properties of the natural extracellular matrix (ECM), promote monocyte recruitment and their polarization toward the regenerative M2 phenotype [29,53].

Upon tissue damage, monocytes are rapidly recruited to the affected tissue, where they can differentiate into tissue macrophages or dendritic cells. The observed results suggest that monocyte activation by xenogenic collagen matrices does not significantly involve PGE2. Although the changes observed for MLCM and VSCM are significant, they may not be biologically prominent. However, the increase in TXB2 production by monocytes stimulated with BLCM and VSCM is significant in magnitude and may hold biological relevance. Similarly, the macrophage stimulation data suggest that these matrices have lower biocompatibility. The differencing patterns of PGE2 and TXB2 responses to xenogenic collagen matrices by monocytes can be attributed to their distinct time courses. TXB2 synthesis is immediate and depends on cyclooxygenase-1 (COX-1) activity, whereas PGE2 synthesis is delayed and depends on COX-2 activity. These apparent COX-isotype dependencies of TXB2 and PGE2 synthesis can be explained by differences in the affinities of TXA synthase and PGE synthase for the common substrate (PGH2) [54]. The non-cross-linked monolayer scaffold demonstrates the lowest potential to stimulate PGE2 and TXB2 production and likely exhibits the highest biological compatibility. Wound healing and soft and hard tissue regeneration can be enhanced by injectable PRF (iPRF). In vitro experiments suggest that platelet derivatives may play a crucial role in inducing a dynamic M1/M2 balance and promoting a timely M1–M2 shift, as observed with the M2-featured macrophages used in the present study [55]. The use of iPRF as a modifying factor, providing additional signals to surrounding cells, appears to interact with MLCM. This interaction modified the responses of the collagen matrix, particularly in macrophages, leading to significantly increased concentrations of PGE2 and TXB2, while being accompanied by a significant decrease in PGE2 production in monocytes.

In the early phase of wound repair, infiltrating monocytes and resident macrophages are activated and primarily acquire a pro-inflammatory (M1) phenotype [56]. These cells phagocytose microbes, scavenge dead cells and cellular remnants, and, upon activation, produce pro-inflammatory mediators such as IL-1, IL-6, and TNFα [56,57].

Later in the healing process, macrophages transition from a pro-inflammatory M1 phenotype to a reparative M2 phenotype. M2 macrophages express anti-inflammatory mediators, such as the IL-1 receptor antagonist and IL-10, as well as growth factors like TGFβ and IGF1, which promote fibroblast proliferation and angiogenesis [58,59]. This M1–M2 transition is critical for resolving inflammation and orchestrating tissue repair [60]. The observed differences in MLCM + iPRF effects—specifically, the inhibition of PGE2 in monocytes and its activation in macrophages—most likely result from the distinct biological functions of these cell types.

Cross-linked (VSCM) and bilayered (BLCM) collagen matrices in the presence of iPRF more strongly affected monocyte and macrophage activity, resulting in an increased level of PGE2 and TXB2 compared with the monolayer matrix (MLCM). The early rise in PGE2 and TXB2 in response to iPRF combined with collagen matrices likely reflects a necessary and beneficial pro-inflammatory phase of regeneration [61]. However, sustained elevated levels can impede the healing process [62]. Without observations showing a decrease in these levels over time, the risk of chronic inflammation cannot be ruled out.

The net balance of the interaction can be neutral in biological systems, but this remains to be fully evaluated. In physiological conditions, macrophages are a major source of PGE2 during inflammation [63,64]. They possess receptors for this eicosanoid and respond to it [65]. The PGE2 produced can regulate cytokine synthesis in an autocrine manner, such as IL-6. This effect is specifically linked to the activation of COX-2 and not COX-1 [66]. IL-6 signaling is then responsible for the transition to a reparative environment [67,68]. These findings suggest that the interaction of iPRF with MLCM may stimulate the regenerative process. In contrast, BLCM and VSCM do not significantly interact with iPRF, as the responses to these biomaterials co-incubated with iPRF did not change significantly.

TXA2 is a known vasoconstrictor that is also activated during tissue injury and inflammation. Due to its instability under experimental conditions, its stable hydrolysis product, TXB2, is commonly used as a marker molecule [50] for COX-1 activity, which mediates TXA2 production in most cells [69]. In the applied experimental model, the most significant changes in the TXB2 levels in monocyte and macrophage cultures were observed in the co-cultures with BLCM and VSCM with the highest changes in macrophages, exceeding a two-fold increase. TXB2 levels were not affected by MLCM in macrophages, BLCM in monocytes, or iPRF itself. The activity of TXA2 in wound healing may be related to its role in inducing blood flow recovery through platelet activation and stimulation of angiogenesis-promoting factors [70]. It may also contribute to the synthesis of the pro-inflammatory cytokine intereukin-6 (IL-6) and PGE2 as well as the inhibition of the expression of the anti-inflammatory macrophage marker CD206 [71]. However, clinical applications of iPRF have demonstrated positive therapeutic effects [72].

Based on the findings of the present study, it can be suggested that the tested collagen matrices interact with monocytes/macrophages, with the non-cross-linked monolayer scaffold demonstrating the highest level of compatibility. iPRF does not seem to significantly influence monocyte and macrophage function in the environment of the studied collagen matrices.

## 4. Materials and Methods

### 4.1. Matrices Examined

In this study, three FDA-approved xenogeneic collagen matrices, deemed suitable as scaffolds for soft tissue augmentation, were obtained (Mucoderm^®^ (MLCM) from Botiss Medical GmbH (Berlin, Germany), Fibro-Gide^®^ (VSCM) from Geistlich Pharma AG (Wolhusen, Switzerland), and Mucograft^®^ (BLCM) from Geistlich Pharma AG (Wolhusen, Switzerland)). A detailed characterization of the collagen matrices examined in the study is provided in Table 3.

### 4.2. Cell Culture and Treatment

The experiments were conducted on human monocytes and macrophages derived from the THP-1 cell line. The cells were cultured in Roswell Park Memorial Institute (RPMI) 1640 medium (cat. CC142-0500, GeneDireX, Taoyuan, Taiwan) supplemented with 100 IU/mL penicillin and 10 µg/mL streptomycin (cat. CC502-0100, GeneDireX, Taoyuan, Taiwan) and 10% fetal bovine serum (FBS, cat. F7524, Merck KGaA, Darmstadt, Germany). The cultures were maintained in a humidified atmosphere at 37 °C with 5% CO_2_, with the medium refreshed every 48 h. Prior to the experiment, THP-1 cells were seeded into culture flasks at an initial density of 1.5 × 10^5^ cells per well (Starlab International, Hamburg, Germany). Differentiation of THP-1 cells into macrophages was induced by treating the cells with 100 nM phorbol 12-myristate 13-acetate (PMA) for 24 h (cat. P1585, Merck KGaA, Darmstadt, Germany). The matrix samples were cut into 0.5 cm × 0.5 cm pieces and rehydrated with injectable platelet-rich fibrin (iPRF), prepared from a single volunteer donor from the research group. The iPRF donor was a healthy, non-smoking young adult (28 years of age) with no blood disorders, infections, or addictions to drugs or alcohol and who was not taking any medications that could negatively affect platelets. A total of 20 mL of peripheral venous blood was collected via sterile venipuncture in two 10 mL iPRF tubes. These tubes were immediately centrifuged using a DUO Quattro centrifuge (Process for iPRF, Nice, France) at a speed of 700 rpm for 3 min, following Choukroun’s protocol [14]. After centrifugation, the collected iPRF samples were promptly applied to the matrices.

In the first experiment, THP-1 cells were cultured for 48 h in complete medium in the presence of the tested materials (MLCM, MLCM + iPRF, VSCM, VSCM + iPRF, BLCM, and BLCM + iPRF). The control group was incubated with complete medium for 48 h. After incubation, the cells were collected, and pellets were obtained by centrifugation (800× *g*, 10 min). In the second experiment, THP-1 macrophages were cultured for 48 h under the same conditions and using the tested materials as described in the first experiment. After incubation, the cells were collected by scraping, and pellets were obtained by centrifugation (800× *g*, 10 min).

The samples were frozen at −80 °C for further analysis. Proteins were extracted using RIPA lysis buffer (cat. 89900, Thermo Fisher Scientific, Waltham, MA, USA), and protein concentration was measured using the Micro BCA Protein Assay Kit (Thermo Scientific, Waltham, MA, USA). The supernatants were transferred to new tubes and stored at −80 °C until further analysis, including the measurement of prostaglandin E2 (PGE2) and thromboxane B2 (TXB2) concentrations using the ELISA method. Differentiation of THP-1 monocytes into THP-1 macrophages (indicating THP-1 monocyte activation) was evaluated using flow cytometry.

### 4.3. Protein Concentration

Protein concentration was determined using the Micro BCA™ Protein Assay Kit (cat. 23225, Thermo Scientific, Waltham, MA, USA) and measured spectrophotometrically (UVM340, ASYS). This kit is designed for the colorimetric detection and quantification of total protein, optimized for dilute protein samples (0.5–20 µg/mL). Based on an adaptation of the Micro BCA™ Protein Assay Kit, it utilizes bicinchoninic acid (BCA) as the detection reagent for Cu^+1^ ions. These ions are generated when Cu^+2^ is reduced by proteins in an alkaline environment. The reaction produces a purple-colored complex formed by the chelation of two BCA molecules with one cuprous ion (Cu^+1^), which is highly water-soluble and exhibits strong absorbance at 562 nm. This provides a highly sensitive colorimetric protein assay suitable for both test tube and microplate formats.

### 4.4. Differentiation of THP-1 Cells into Macrophages: Flow Cytometry Analysis

Differentiation of THP-1 monocytes to macrophages was determined by staining CD14 and CD68 markers with monoclonal antibodies and evaluated by flow cytometry. The cells were stained with FITC Mouse Anti-Human CD14 or Alexa Fluor^®^ 647 Mouse Anti-Human CD68 (BD Pharmingen, San Diego, CA, USA). The cells were stained according to the manufacturer’s protocol. Briefly, the cells were collected (macrophages by scrapping), washed with phosphate-buffered saline (PBS) supplemented with 2% bovine serum albumin (BSA, Sigma-Aldrich, St. Louis, MO, USA), and stained in PBS with BSA with an appropriate antibody for 1 h. Next, the cells were washed in PBS, resuspended in PBS, and analyzed in a flow cytometer (CytoFlex, Beckman Coulter, Brea, CA, USA).

### 4.5. Verification of Collagen-Matrix-Induced Initiation of the Inflammatory Response in Macrophages

THP-1 macrophages were cultured for 48 h under the same conditions and with the tested materials as described in the first experiment. Following incubation, the cells were collected as previously described, and protein concentrations were measured using the Micro BCA Protein Assay Kit (Thermo Scientific, Rockford, IL, USA). The concentrations of PGE2 and TXB2 were determined using the ELISA method, while COX-1 and COX-2 expression levels were analyzed using an immunocytochemical method with a confocal microscope.

#### 4.5.1. COX-1/2-Mediated Production of PGE2 and TXB2

The activity of the cyclooxygenases COX-1 and COX-2 was investigated by quantifying their products (prostaglandin E2 (PGE2) and thromboxane B2 (TXB2)). Cells were incubated for 48 h with MLCM or MLCM + iPRF, VSCM or VSCM + iPRF, and BLCM or BLCM + iPRF, as described above. The concentration of PGE2 (cat. EH4233, FineTest, Wuhan, China) and TXB2 (cat. EP0162, FineTest, Wuhan, China) was measured spectrophotometrically using an enzyme immunoassay kit according to the manufacturer’s protocol.

#### 4.5.2. Imaging of Cyclooxygenase-1 and Cyclooxygenase-2 Expression

Cells were cultured with collagen matrices on microscope slides following the previously described protocol. After cultivation, the cells were rinsed with PBS and fixed in 4% buffered formalin for 15 min at room temperature. Following fixation and subsequent PBS washes, the cells were permeabilized using a 0.5% Triton X-100 solution in PBS. After additional PBS washes, the cells were incubated overnight at 4 °C with primary mouse anti-COX-1 and anti-COX-2 antibodies (cat. ab81296 and cat. ab62331, Abcam, Cambridge, UK) at a 1:50 dilution. The cells were then washed and incubated with a secondary anti-rabbit IgG FITC-conjugated antibody, diluted 1:60 (cat. F0382, Sigma-Aldrich, Poznan, Poland) in antibody diluent (Agilent Dako, Santa Clara, CA, USA), for 30 min at room temperature. After further PBS washes, the cells were stained with Hoechst 33258 for 30 min at room temperature.

The samples were analyzed using a confocal microscope (FV1000 confocal system with an IX81 inverted microscope, Olympus, Hamburg, Germany). Three-channel acquisition and sequential scanning were applied to achieve the optimal resolution for Hoechst 33258 and FITC fluorescence signals. Fluorescent images were merged with transmitted light images for enhanced visualization.

#### 4.5.3. Western Blot Analysis of Cyclooxygenase-1 and Cyclooxygenase-2 Expression

Electrophoretic protein fractionation was performed on an 8–16% polyacrylamide gel (cat. GSM00660, GenSignal, Poznań, Polska) at 10 μg protein/well. Prior to electrophoresis, the samples were heated at 90 °C for 5 min with 2-mercaptoethanol (Sigma-Aldrich, St. Louis, MO, USA) and Laemmli buffer (Laemmli Sample Buffer, Bio-Rad, Feldkirchen, Germany). The proteins were transferred using wet transfer to a 0.2 μm PVDF membrane (Thermo Fisher Scientific™, Waltham, MA, USA). Before incubation with antibodies, the membranes were placed in a blocking buffer (5% skimmed milk) for 60 min. The expression of COX-1 (ab81296) and COX-2 (ab62331) proteins was detected using antibodies from Abcam (Cambridge, UK). The expression of the reference protein GAPDH (ab8245) was detected. HRP-labeled anti-mouse (ab6789) and anti-rabbit (ab205718) secondary antibodies from Abcam (Cambridge, UK) were used. The membranes were developed using an ECL Advance Western Blotting Detection Kit (GE Healthcare, Chicago, IL, USA), and the bands were visualized using a ChemiDoc XRS+ molecular imager (Bio-Rad, Hercules, CA, USA) and analyzed using ImageLab 6.1 software (BioRad, Feldkirchen, Germany)

### 4.6. Statistical Analysis

Statistical analysis of the results was performed using Statistica 10 software (StatSoft, Poland). Data are expressed as the arithmetic mean ± standard deviation (SD). The distribution of variables was assessed using the Shapiro–Wilk W-test. Since most distributions deviated from normality, nonparametric tests were applied for further analyses. The results were evaluated using the Mann–Whitney U test, with the significance level set at *p* < 0.05.

## Figures and Tables

**Table 1:** The effect of collagen matrices (MLCM, VSCM, and BLCM) on the activation of THP-1 monocytes.

Fluorescence Intensity			
CD14 (% of Positive Cells)	CD68 (% of Positive Cells)		
99.96%	2.8%	THP-1	MLCM
98.37%	79.94%	THP-1 + PMA	
99.92%	2.5%	THP-1	VSCM
99.72%	78.26%	THP-1 + PMA	
99.98%	2.3%	THP-1	BLCM
98.41%	80.43%	THP-1 + PMA	

**Table 2:** Immunofluorescence score for COX-1 and COX-2 protein expression based on staining intensity in in vitro cultures of THP-1 monocytes and macrophages.

Fluorescence Intensity			
COX-1 (Positive Cells) Monocytes/ Macrophages	COX-2 (Positive Cells) Monocytes/ Macrophages		
+/+	+/++	THP-1	Control
+/+	+/++	THP-1	MLCM
+/+	+/++	THP-1 + PMA	
+/+	+/++	THP-1	VSCM
+/+	+/++	THP-1 + PMA	
+/+	+/++	THP-1	BLCM
+/+	+/++	THP-1 + PMA	

Expressions are described as follows: +, positive; ++, moderate. Scoring was performed by two independent researchers without knowing the sample’s description.

**Table 3:** Detailed data on collagen matrices used for testing.

Collagen Matrix	Origin	Scaffolding Characteristics	Effects	Reference
Mucoderm^®^ (Botiss medical GmbH, Berlin, Germany) monolayer collagen matrix (MLCM)	Porcine dermis	Monolayer scaffold collagen types I and III elastin non-cross-linked	Promotes the proliferation of fibroblasts and endothelial cells and supports rapid revascularization	[3,73]
Fibro-Gide^®^ (Geistlich Pharma AG, Wolhusen, Switzerland) volume-stable collagen matrix (VSCM)	Porcine dermis	Monolayer scaffold collagen types I and III elastin volume-stable cross-linked	Promotes the ingrowth of fibroblasts, matrix biosynthesis, tissue integration, and angiogenesis	[12,74]
Mucograft^®^ (Geistlich Pharma AG, Wolhusen, Switzerland) bilayer collagen matrix (BLCM)	Porcine peritoneum (compact layer) Porcine dermis (porous layer)	Bilayer scaffold (compact and porous layers) collagen types I and III non-cross-linked	Compact layer acts as a barrier and provides stability Porous layer stabilizes the blood clot and promotes cell ingrowth, tissue integration, and angiogenesis	[11,75]

## Data Availability

Data supporting the reported results can be found in the Repository of the Pomeranian Medical University in Szczecin.
